# Supplementary material for: Extracellular vesicles from CLEC2-activated platelets enhance dengue virus-induced lethality via CLEC5A/TLR2
Source: Nat Commun. 2019 Jun 3;10:2402. doi: 10.1038/s41467-019-10360-4 (PMC6546763; doi:10.1038/s41467-019-10360-4)
Supplement: Supplementary file 2 — Supplementary Information [file 41467_2019_10360_MOESM2_ESM.pdf]

Supplementary information

Extracellular vesicles from CLEC2-activated platelets enhance dengue virus-induced lethality via CLEC5A/TLR2

Sung et al.

Supplementary Figure 1

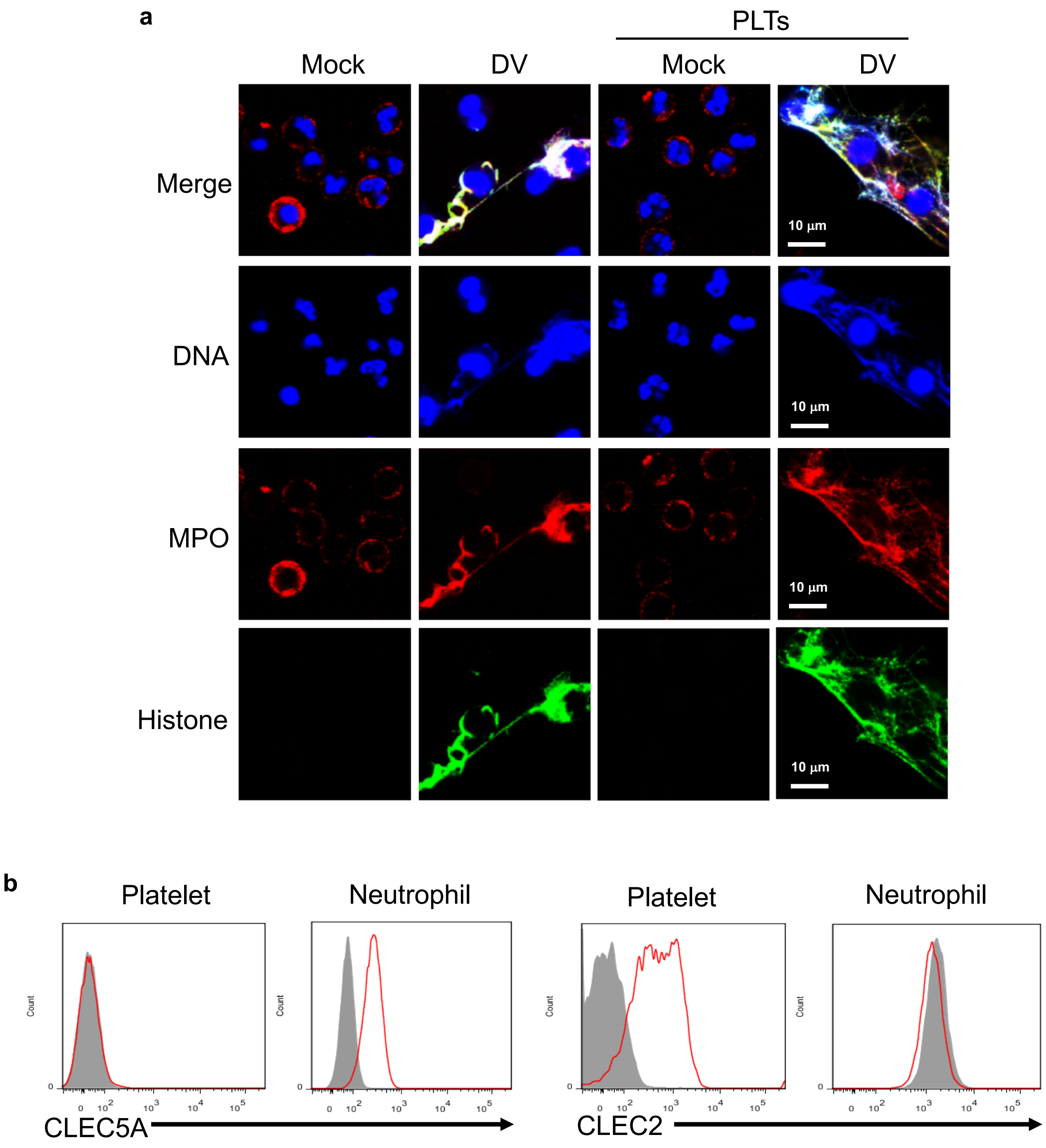

Supplementary Figure 1. Immunofluorescence staining of NET structure and expression levels of CLEC5A, CLEC2 in human neutrophils and platelets. (**a**) DNA, histone, and MPO were detected by Hoechst 33342 (blue), anti-histone antibody (green), and anti-MPO antibody (red), respectively. Scale bar: 10 μm. (**b**) Expression level of CLEC5A and CLEC2 in human platelet and neutrophil were determined by flow cytometry.

Supplementary Figure 2

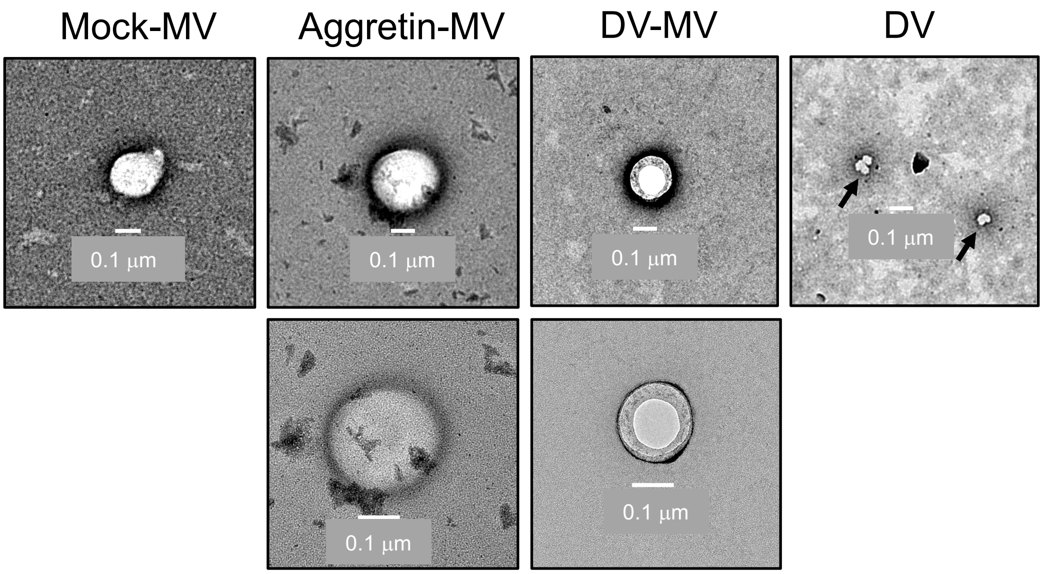

Supplementary Figure 2. Ultrastructure of DV-activated platelets-derived MVs and EXOs. Ultrastructure was observed under transmission electron microscopy. Scale bar: 0.1 μm

Supplementary Figure 3

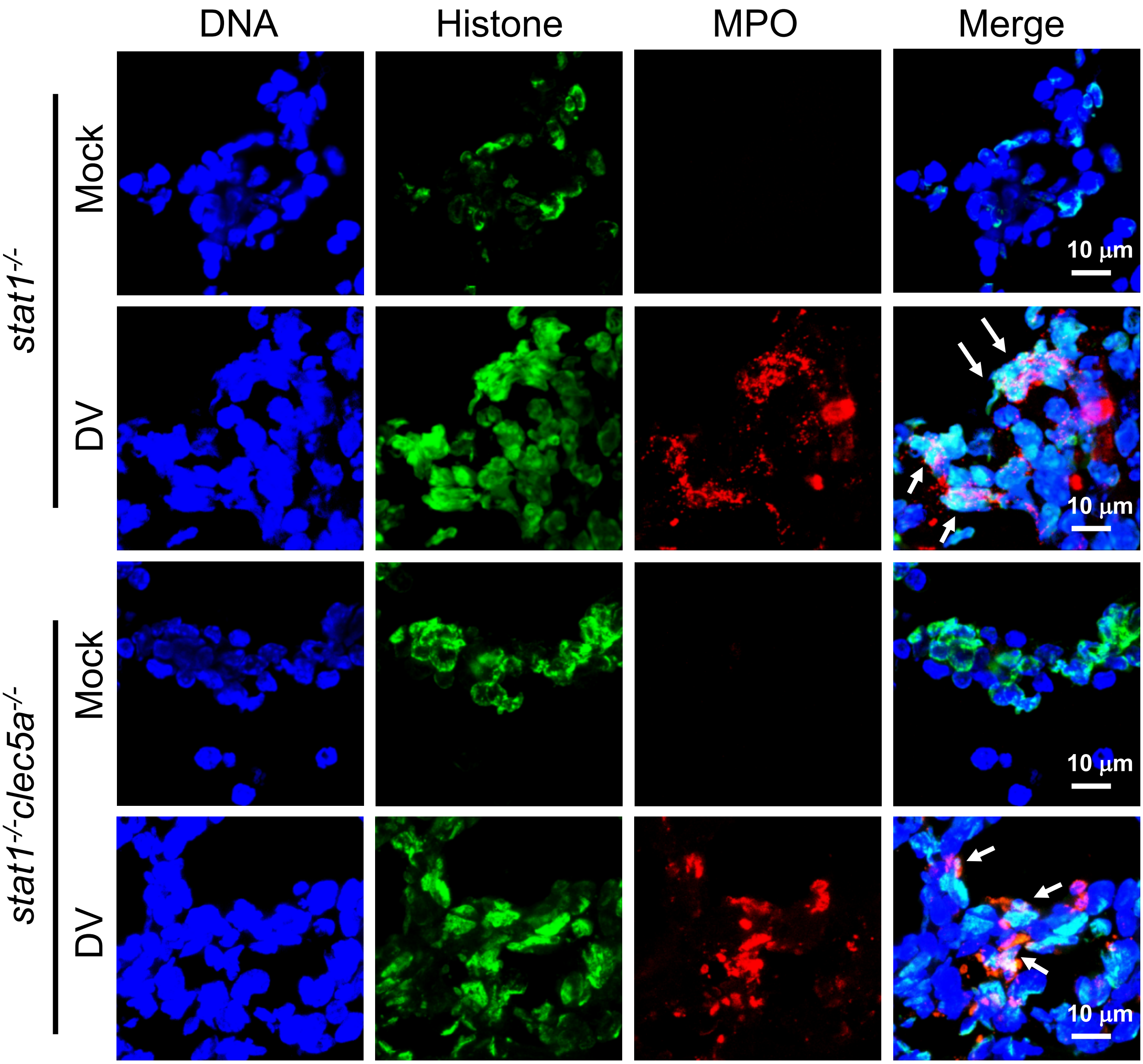

Supplementary Figure 3. Immunofluorescence staining of the NET structure in spleen of DV-challenged mice. *Stat1*$^{-/-}$ and *stat1*$^{-/-}$*clec5a*$^{-/-}$ mice were inoculated with DV (NGC-N, 2x10^5 PFU) via intraperitoneal route. Spleens were harvested and fixed at day 5 post-infection for immunofluorescence staining using anti-MPO mAb (red), anti-histone mAb (green), and Hoechst 33342 (blue). Scale bar: 10 μm

Supplementary Figure 4

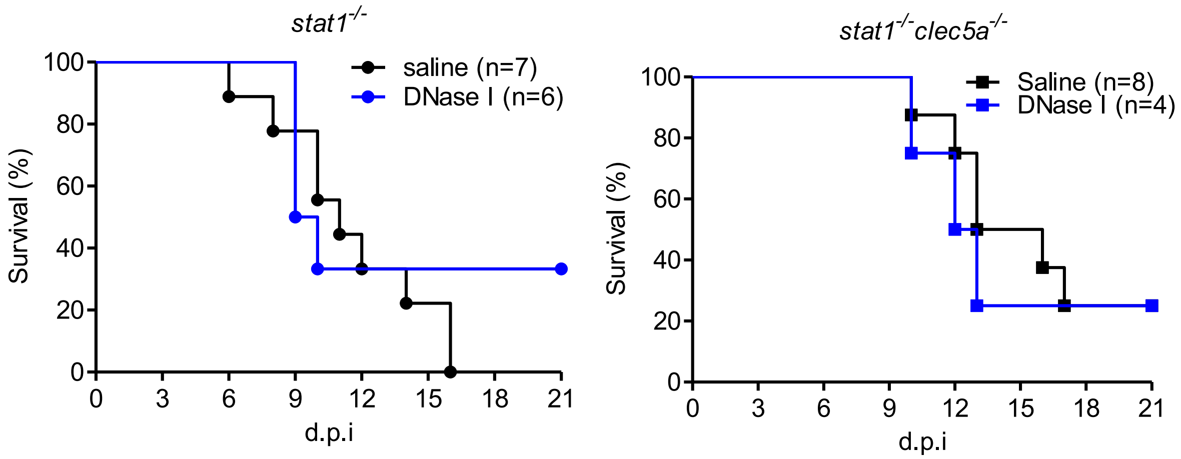

Supplementary Figure 4. DNase I injection rescues the survival rate in DV-challenged *stat1*$^{-/-}$ mice. *Stat1*$^{-/-}$ mice (left panels) and *stat1*$^{-/-}$*clec5a*$^{-/-}$ mice) (right panels) were challenged with lethal dose of DV via intraperitoneal route. DNase I (4 KU) were injected intraperitoneally at day 0, 2, 4, and 6 post infection. Survival rate was measured every day till day 21 post-infection.

Supplementary Figure 5

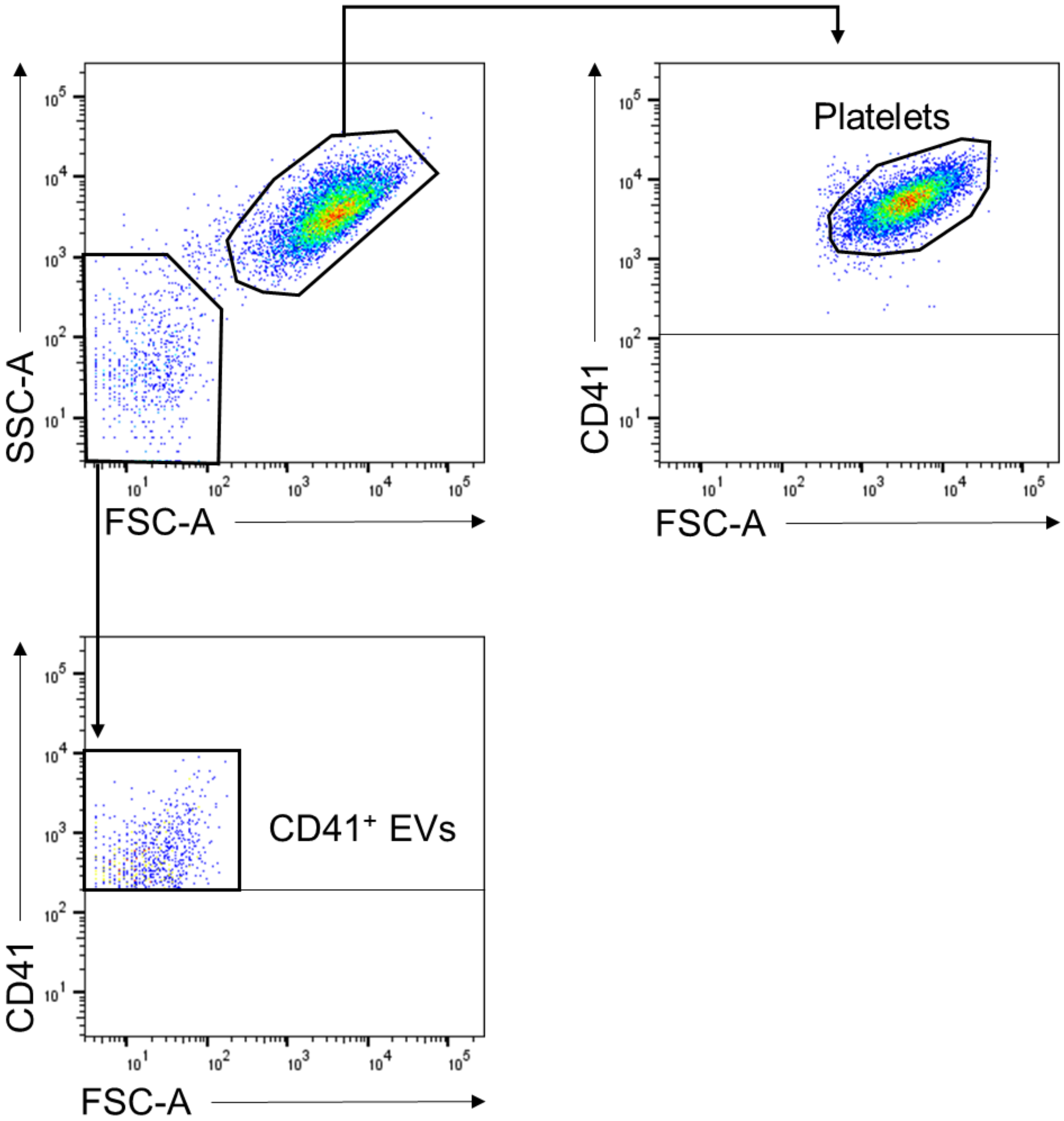

Supplementary Figure 5. Gating strategy for platelet population and CD41⁺ EVs. Gating strategy for human/mouse platelets and CD41+ EVs for Fig. 1a, and Fig. 3a.

Supplementary Table 1

	Protein name	Gene name	Increase/ Decrease	Significantly different compared to mock (Score)
**MVs**	Tubulin beta-1 chain	TUBB1	↑	28.166
	Guanine nucleotide-binding protein G(I)/G(S)/G(O) subunit gamma-3	GNG3	↑	6.0121
	Tribbles homolog 1	TRIB1	↑	5.8874
**EXOs**	Vinculin	VCL	↑	21.874
	Coagulation factor XIII A chain	F13A1	↑	18.076
	Calnexin	CANX	↑	11.591

Supplementary Table 1. Mass spectrometry analysis of aggretin- and DV-activated platelets-derived MVs and EXOs. Proteomic comparison of MVs and EXOs from resting platelets and CLEC2-activated platelets by LTQ Orbitrap XL mass spectrometer. Unique proteins which up expressed in CLEC2-MVs and EXOs are listed.
